# Supplementary material for: Clinical Feasibility Study of Gold Nanoparticles as Theragnostic Agents for Precision Radiotherapy
Source: Biomedicines. 2022 May 23;10(5):1214. doi: 10.3390/biomedicines10051214 (PMC9139134; doi:10.3390/biomedicines10051214)
Supplement: Supplementary file 1 [file biomedicines-10-01214-s001.zip › biomedicines-1729841-supplementary.pdf]

Supplementary Materials

Table S1. Elemental weight proportions of the generated materials.

Element	Air (density: 0.00120479 g/cm³)	Lung, with air (density: 0.26 g/cm³)	Soft tissue (density: 1.00 g/cm³)	Bone (density: 1.85 g/cm³)
H	-	0.103	0.10447	0.064
C	0.000124	0.105	0.23219	0.278
N	0.755267	0.031	0.02488	0.027
O	0.231781	0.749	0.63024	0.410
Na	-	0.002	0.00113	-
Mg	-	-	0.00013	0.002
P	-	0.002	0.00133	0.070
S	-	0.003	0.00199	0.002
Cl	-	0.003	0.00134	-
Ar	0.012827	-	-	-
K	-	0.002	0.00199	-
Ca	-	-	0.00023	0.147
Fe	-	-	0.00005	-
Zn	-	-	0.00003	-
Total	1	1	1	1

Table S2. AuNP material elemental composition. Weight proportions inferred from 'soft tissue' material.

Elements	2.23 mg Au/g of tissue (density: 1.00223 g/cm³)	22.3 mg Au/g of tissue (density: 1.02230 g/cm³)	40 mg Au/g of tissue (density: 1.18290 g/cm³)
H	0.10423903	0.10214227	0.10029312
C	0.23167222	0.22701216	0.22290240
N	0.02482452	0.02432518	0.02388480
O	0.62883257	0.61618369	0.60502848
Na	0.00112748	0.00110480	0.00108480
Mg	0.00012971	0.00012710	0.00012480
P	0.00132703	0.00130034	0.00127680
S	0.00198556	0.00194562	0.00191040
Cl	0.00133701	0.00131012	0.00128640
K	0.00198556	0.00194562	0.00191040
Ca	0.00022949	0.00022487	0.00022080
Fe	0.00004989	0.00004889	0.00004800
Zn	0.00002993	0.00002933	0.00002880
Au	0.00223000	0.02230000	0.04000000
**Total**	**1**	**1**	**1**

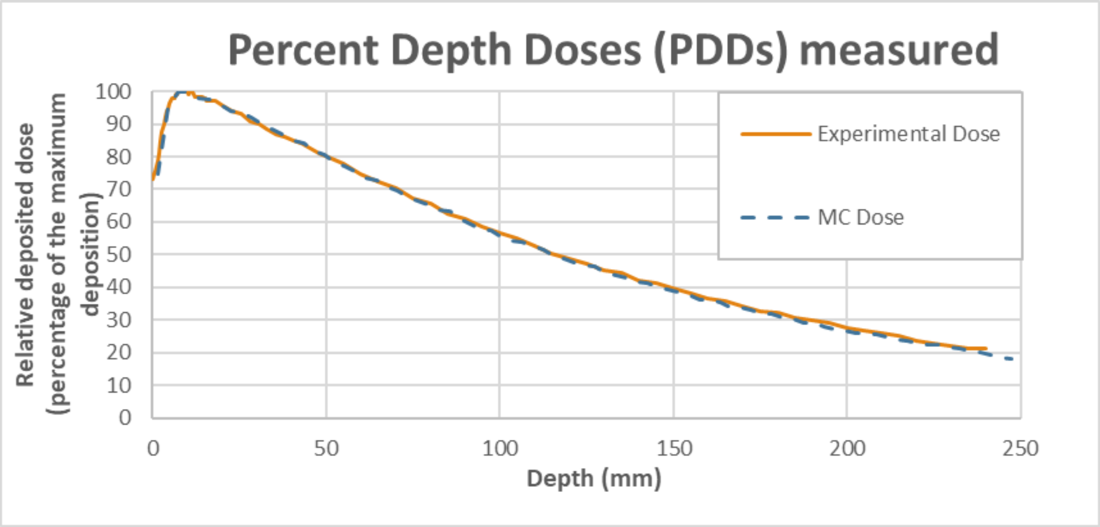

Figure S1. Percent depth dose (PDD) profiles measured in both experimental dosimetry (orange solid line) and Monte Carlo (MC) simulations (blue dashed line).

Table S3. CT data of each standardized material scanned.

Material	Physical density (g/cm^3)	Electronical density (x10^{23} electrons/cm^3)	HU
Lung (inhaled)	0.2	0.634	-829.1
Lung (exhaled)	0.5	1.632	-489.1
Adipose	0.96	3.170	-72.6
Breast (50% adipose/50% glandular)	0.99	3.261	-34.4
Muscle	1.06	3.483	46.7
Liver	1.07	3.516	55.4
Dense bone (800 mg/cm3)	1.53	4.862	1036.1

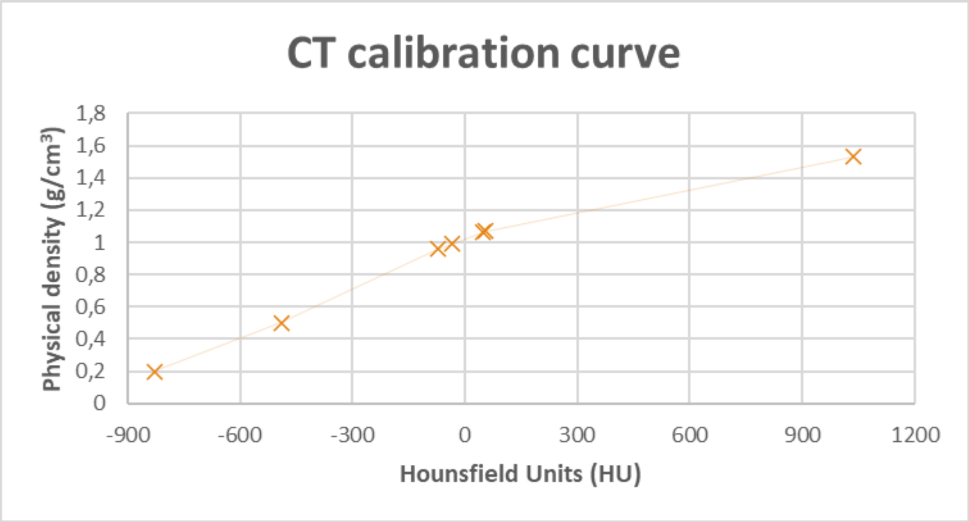

Figure S2. CT calibration curve obtained when representing physical density vs. HU of each material, based on data represented in table S3.

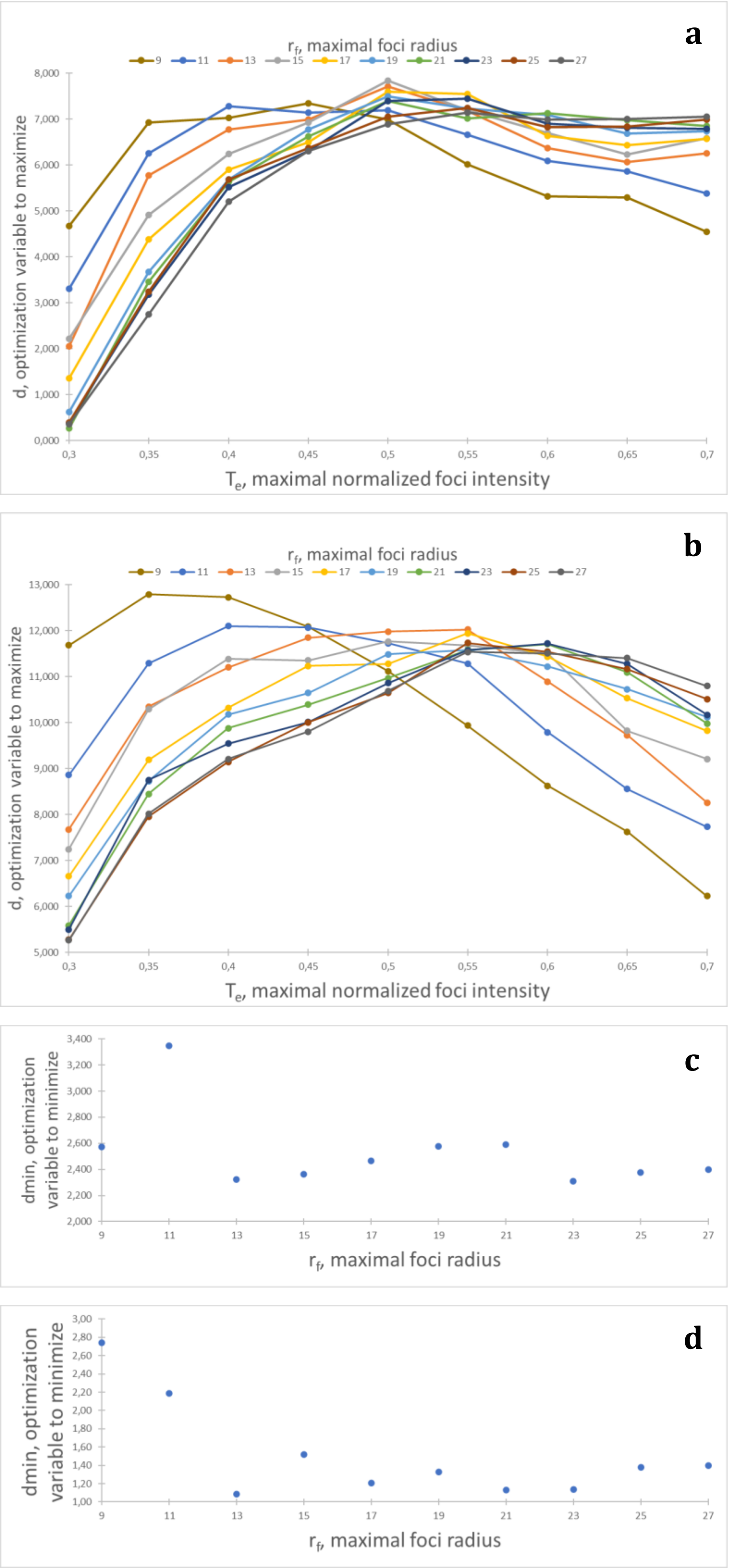

Figure S3. Optimization curves for parameters r_f, T_e. (a, b) First optimization procedure without nanoparticles (a) and with nanoparticles (b). (c, d) Second optimization procedure without nanoparticles (c) and with nanoparticles (d).

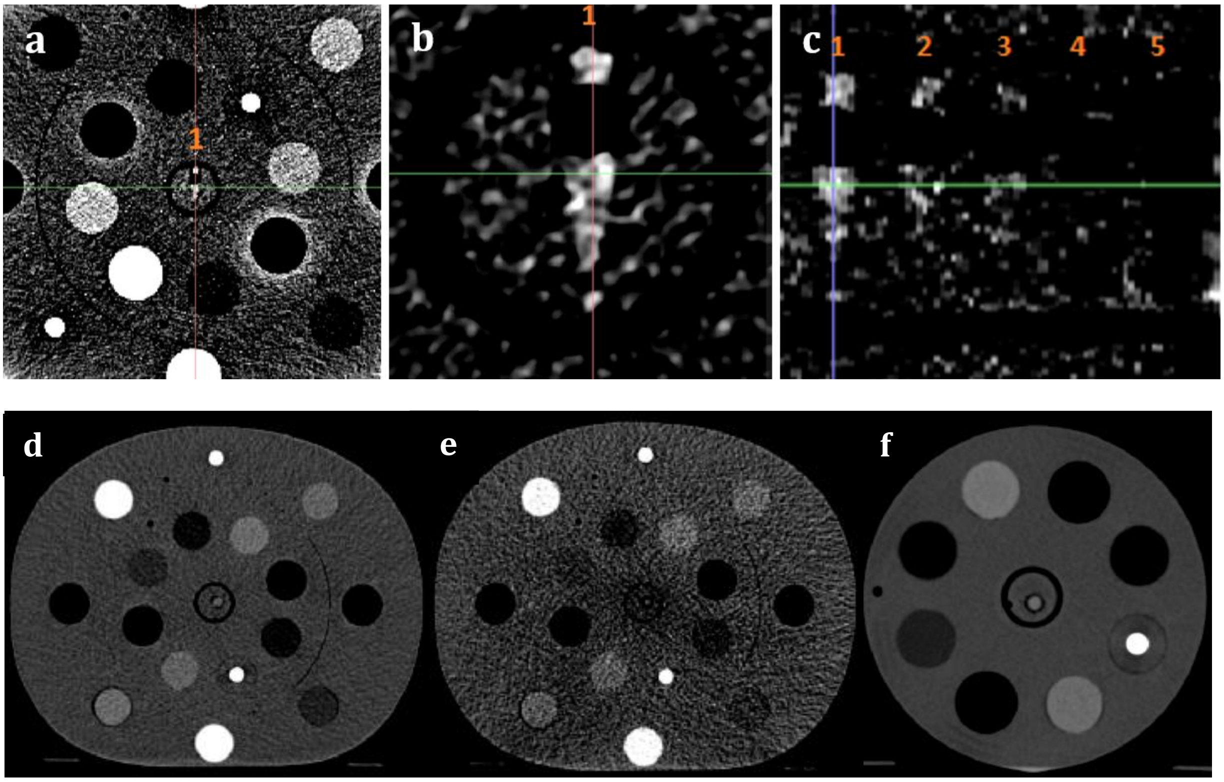

Figure S4. (a, b, c) CT scan images for the seriated AuNP concentrations experiment, in ~0,2 cm^3 tubes. The axes are centered in the radiological concentration. (a) Axial slice, (b) detailed zoom with a narrower window level, and (c) sagittal slice where other concentrations are present. Each vial contains the following: (1) original AuNP concentration of 2,23 mg Au/mL, (2) dilution with 1:2 factor, (3) dilution with 1:4 factor, (4) water, (5) air.

(d, e, f) Axial slices from three different acquisitions of the phantom containing the AuNP vial positioned in the center of the central insert are shown. Window level was adjusted to the same level for all three images – from -60 to 200 HU, and the acquisition energy was 120 keV for all of them. Images correspond to (d) body configuration in phantom, 'wholebody' reconstruction, 5 mm slice thickness; (e) body configuration in the phantom, 'wholebody' reconstruction, 0.6 mm slice thickness, (f) head & neck phantom configuration, 'brain' reconstruction, 3 mm slice thickness; a zoom regarding images (d) and (e) was applied.

Files S1. Immunofluorescence images, available to download at https://hdvirtual.us.es/discovirt/index.php/s/aa7K5aMf7G9i6Fe
